# The Therapeutics of Inflammation

**Published:** 1899-09

**Authors:** Warren B. Hill

**Affiliations:** Milwaukee, Wis.


### The Therapeutics of Inflammation.

BY WARREN B. HILL, M.D., MILWAUKEE, WIS.

Abstract of paper read before the Section of Stomatology, American Medical Association, Columbus, June, 1899.

There is a common ground upon which dentists and doctors may meet, and the therapeutics of inflammatory conditions about the mouth, though a place oftentimes studiously avoided by both professions, should be cultivated in common by both. The rational treatment for all inflammatory processes is to remove the cause, if possible. This usually takes us into the field of surgery, as nearly all the inflammatory processes are of bacterial origin, but there is still left for the therapeutist an opportunity of relieving the pathological conditions present when the other alternative is not to be accomplished or during the time when a diagnosis is being made.

Heat and cold are the two remedies most extensively used for the relief of congestion and pain, and there has been considerable discussion as to which is the more efficacious for this purpose. The up-to-date surgeon advocates, when he appears before a learned body of his professional brethren, the use of cold only, as that impedes the propagation of the germs which he assumes cause the trouble.

In his private practice, however, he allows the use of hot applications and poultices, because it relieves the pain, and nobody will find out how antiquated his practices are in comparison with his precepts. On the other hand, the empiricist adheres strictly to precept and practice to the use of hot applications, because experience has taught him that it alleviates the two prominent symptoms present, engorgement and pain.

For my part, however, it appears that each has a proper place in the therapeutics of inflammation, without violating the laws of reason or repudiating clinical experience.

In the first stage of inflammation, when there is dilatation of the afferent blood vessels and an increase in the rapidity of the flow of blood, cold applied to the part will contract these vessels and prevent the subsequent engorgement, and in this manner pain may be avoided. On the other hand, when the engorgement is already present and the blood status has supervened, then the application of heat will dilate the afferent vessels, relieve the engorgement and alleviate the pain.

This same principle applied to internal medication will also be useful in the relief of these symptoms. The immediate indications in the treatment of these conditions are the relief of pain. In true inflammatory processes, pain is caused by the engorgement of the blood vessels and the impingement upon the nerve filaments by the consequent exudate. The rational method, therefore, of relieving it is to reduce the arterial tension. This may be done by dilating the peripheral vessels, either by the use of diaphoretics, cardiac depressants, or counter-irritants.

Arterial tension is also reduced by the use of hydrogogue cathartics, and congestions about the head are particularly benefited by the use of cholagogues. On the other hand, opiates, by checking alimentary secretion and increasing the blood supply to the head, not only fail to be useful, but are contra-indicated, except when given in the form of Dover's powder, which acts as a powerful diaphoretic and relieves the congestion. In painful affections of an æsthenic type, such as in neuralgias caused by the faulty nutrition of the nerve centers, they act promptly and well. In treating inflammation about the mouth I think the following hints will be of service: First, a powerful purge, such as calomel in ten grain doses, should be given, followed by a saline cathartic. Second, a coal-tar analgesic acting upon the skin, such as antipyrin, in from five to ten grain doses, or, in people of rheumatic tendencies, salol and phenacetin in five grain doses each, or acetanilid and salicylate of soda in similar doses. If, on account of the condition of the patient, these heart-depressing coal-tar derivatives may not be deemed advisable, Dover's powder in five to ten grain doses may be substituted.

This treatment is not calculated in any way to remove the cause of the malady, but rather to mitigate the pathological conditions present during the time of diagnosis and the completion of the surgical procedure. In inflammation, especially in the bony cavities about the mouth, such as pulpitis, this treatment will be found to be particularly valuable, as a considerable time often elapses before an accurate diagnosis can be made. The rationale of this treatment is apparent as the lowering of the arterial tension by cathartics and diaphoretics not only prevents any further exudate and consequent pain, but also promotes absorption, while the coal-tar derivatives have specific analgesic properties. Illustrative of this point I wish to relate a case. Mr. X., age forty-five, a laborer, was attacked with severe headache. A physician was consulted who pronounced it neuralgic in its nature. The patient was told to consult a dentist, who extracted one or two teeth in the neighborhood of the most painful portion of the jaw, from which the pain seemed to start. This afforded no relief. The dentist was asked to remove the one remaining tooth, which he refused to do, because it was perfectly sound. He was referred back to the doctor, who was convinced that his diagnosis was wrong and proceeded to treat the case symptomatically, as he had no basis for a diagnosis.

Morphine was given in one-sixth grain doses, which was increased until the patient received one-third of a grain every three hours, the result being that the pain increased to such an extent that he could not lie down at all. At the same time, he could not stay awake long enough to stand up. When in this pitiable condition, I was called to see him. I made a diagnosis of a circumscribed inflammation within a bony cavity and too much morphine, and prescribed ten grains of calomel and ten grains of jalap to be taken in one dose, followed in four hours by one ounce of epsom salts. The relief was remarkable, and to my mind strengthened the diagnosis. Dr. G. V. I. Brown was called in consultation and a careful examination was made. Percussion elicited the fact that an inflammatory process was going on in the pulp of this apparently sound tooth. Dr. Brown removed the cause and an immediate recovery followed.

The conclusions that I wish to draw from this case are: First, that patients should not be sent from doctor to dentist and back again when a consultation is possible; second, that we should not take such a radical view of surgical procedure as the only method of curing inflammatory processes, as to prevent our using all possible means for the relief of the patients during the time when a diagnosis is made and the surgical treatment instituted; third, that we should not resort to the promiscuous use of opiates or any other analgesic as a temporary measure when the pathological conditions may be treated rationally; fourth, having made our patients comfortable we should take plenty of time to make an absolute and accurate diagnosis, thereby saving the patient the annoyance of undergoing unnecessary and painful operations, and possibly preserving for him his teeth or other necessary organs.

